# Present and Future Post-marketing Drug Safety Assessment in Japan: A PMDA Perspective

**DOI:** 10.1007/s43441-026-00964-6

**Published:** 2026-04-24

**Authors:** Takashi Waki, Shinya Watanabe, Takashi Ando, Kazuhiro Kajiyama, Koichi Fukuda, Eiko Iwasa, Yukari Iwasaki, Masao Iwagami, Taihei Tanaka, Daisuke Maeda, Yoshiaki Uyama

**Affiliations:** 1 Office of Pharmacovigilance I, Pharmaceuticals and Medical Devices Agency, Tokyo, Japan; 2 Office of Pharmacovigilance II, Pharmaceuticals and Medical Devices Agency, Tokyo, Japan; 3 Office of Regulatory Science Research, Center for Regulatory Science, Pharmaceuticals and Medical Devices Agency, Tokyo, Japan; 4 Center for Regulatory Science, Pharmaceuticals and Medical Devices Agency, 3-3-2 Kasumigaseki, Chiyoda-ku, Tokyo, 100-0013 Japan

**Keywords:** Pharmacovigilance, Drug safety, Regulatory decision, Real-world data, Pharmacoepidemiology, Individual case safety reports, Artificial intelligence, New methods and technology

## Abstract

For pharmacovigilance, the Pharmaceuticals and Medical Devices Agency in Japan has utilized real world data (RWD) from multiple sources, including individual case safety reports, and medical information databases that capture routinely collected data from clinical practice. These RWD have their own characteristics with advantages and disadvantages. In this commentary, we describe current and future direction of post-marketing drug safety assessment in Japan.

The Pharmaceuticals and Medical Devices Agency (PMDA) in Japan plays a key role in pharmacovigilance via its so-called “Safety Triangle”, which consists of regulatory review for approval, post-marketing safety measures, and relief services for adverse health effects [1]. PMDA currently employs two primary and complementary approaches to derive scientific evidence for regulatory decision-making on drug safety in addition to the review of literature and safety measures taken by foreign regulatory agencies: (i) evaluations based on reports, such as individual case safety reports (ICSRs) and periodic benefit-risk evaluation reports; and (ii) pharmacoepidemiological studies conducted by PMDA using medical information databases including electronic health records and administrative claims data. This paper presents the PMDA perspective on current and future direction of post-marketing drug safety assessment in Japan.

In ICSR review, PMDA routinely evaluates detailed case information, including clinical course, comorbidities, and laboratory test results. A distinctive feature of Japanese ICSRs is their high level of completeness [2], which allows even descriptive reviews to provide clinically meaningful insights into the relationship between a drug and an adverse reaction. Since 2014, PMDA has also initiated to conduct pharmacoepidemiological studies utilizing medical information databases under the Medical Information for Risk Assessment Initiative (MIHARI) [3]. For post-marketing drug safety assessment, the PMDA has routinely utilized the Medical Information Database Network (MID-NET^®^) and National Database of Health Insurance Claims and Specific Health Checkups of Japan (NDB) [4–6]. MID‑NET^®^ is a PMDA‑operated medical information database established under a government project comparable to the US Sentinel Initiative [7] and DARWIN EU [8]. Unlike the NDB, it includes detailed electronic health record data, such as laboratory test results, enabling more in‑depth pharmacoepidemiological evaluations despite a smaller patient population. To strengthen the functionality of MID-NET^®^, the PMDA has recently developed a rapid and continuous monitoring tool with automated function within MID-NET^®^ for safety signal monitoring of a new drug [9, 10]. With this function, results comparing risks between exposure and control groups across 46 outcomes, including laboratory parameters related to liver, kidney, hematological systems, biomarkers, and electrolytes, can be generated within 15 days.

The PMDA currently faces two main challenges in post-marketing drug safety assessment (Fig. 1). The first is the year-by-year rise in numbers of ICSRs due to increasing numbers of approved drugs in the market, an aging society, and growing awareness of the adverse drug reaction reporting system and its computerization. The second is the efficient integration of multiple assessment approaches, including analysis of ICSRs and pharmacoepidemiological studies based on medical information databases. It has been reported that ICSRs may be useful in detecting some types of safety signals, such as idiosyncratic reactions (e.g., anaphylaxis reaction) and events related to the pharmacological effects of the drug, but not in detecting events related to an increased frequency of “spontaneous” disease (e.g., thromboembolic complications caused by oral contraceptives) [11]. More quantitative approaches such as pharmacoepidemiological studies based on medical information databases may evolve the scientific approach to post-marketing drug safety assessment. Such approaches can leverage rich patient-level data, including comorbidities, concomitant medications, prescriptions during follow-up, and laboratory test results, and, by incorporating appropriate comparator groups and adjusting for confounding factors, enable the estimation of relative risks of outcome occurrence with greater statistical power, leading to better scientific evidence.

**Fig. 1:**
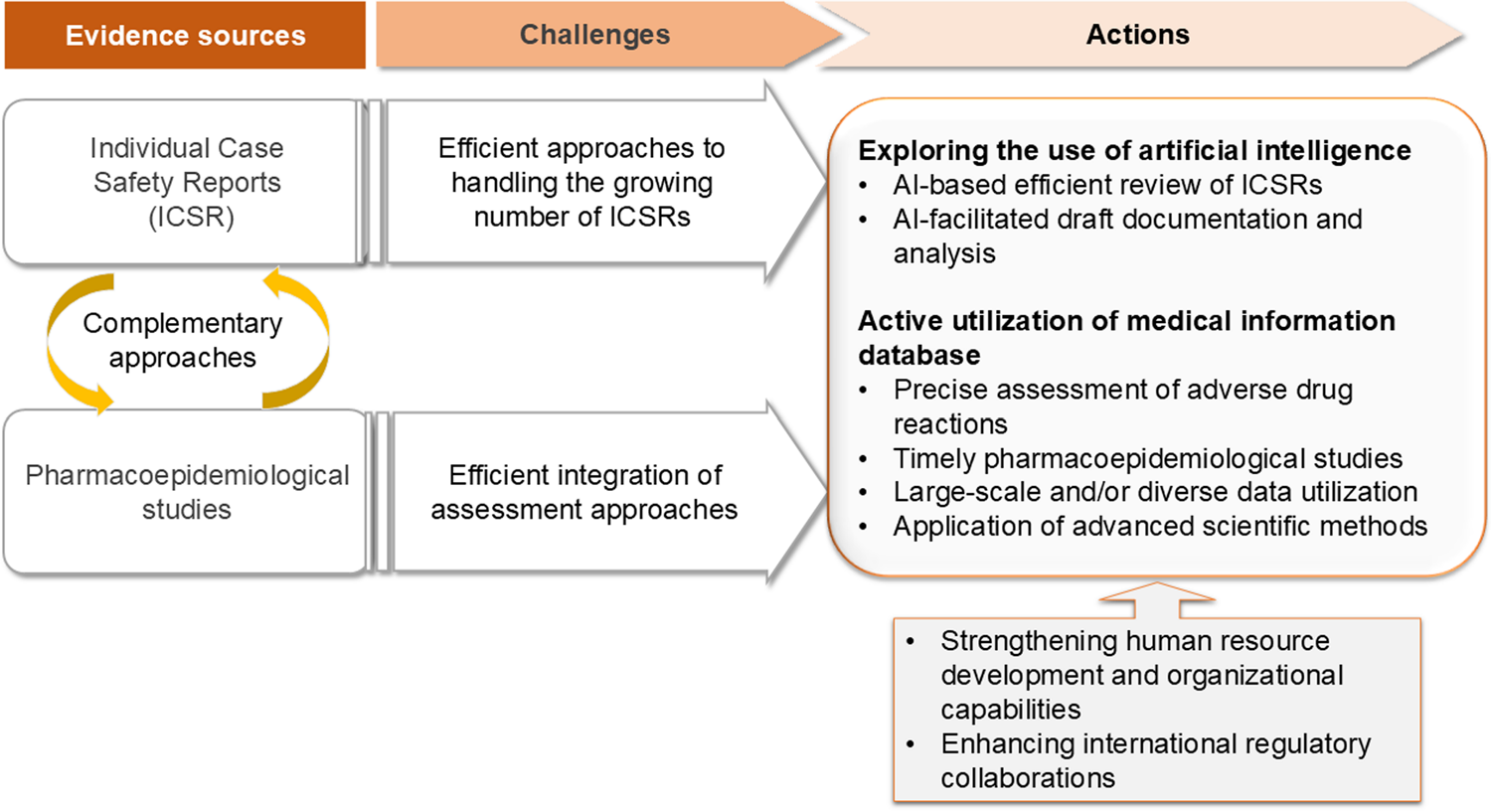
Challenges and actions toward the next stage of post-marketing drug safety assessment in Japan

To appropriately address these challenges, we have started exploring the application of artificial intelligence (AI) technologies in drug safety assessment within PMDA, as described in the recently published PMDA action plan [12]. In particular, the utilization of AI may increase efficiency in reviewing ICSRs by automatically identifying well-informed ICSRs with sufficient information for causality assessment, duplicated ICSRs, and reports with new drug risks. Generative AI is also expected to be utilized to summarize information necessary for regulatory actions, such as information from drug labeling and published literature. With respect to the use of AI in pharmacoepidemiological studies, its application is envisaged for drafting study protocols or reports and for creating and logically validating draft analysis programs. Through these activities, we aim to gain insights into challenges related to human oversight and accountability, ultimately improving the efficiency of drug safety assessment in ICSR review and pharmacoepidemiological studies, while establishing appropriate management approaches such as clearly defined roles and responsibilities, human-in-the-loop review processes, and documentation to ensure transparency and traceability.

In addition, PMDA will more actively utilize medical information databases such as MID-NET^®^ and NDB, together with ICSRs for post-marketing drug safety assessment [13]. More precise evaluations of the time-to-onset of adverse drug reactions and their effect modifiers will contribute to the formulation of more concrete safety warnings and enhance the feasibility of appropriate responses in clinical practice, thereby promoting the proper use of drugs. In order to conduct pharmacoepidemiological studies in a timely and efficient manner, efforts are also underway to modularize analysis programs into reusable components for standard analytical steps of typical study designs (e.g., data cleaning, inclusion and exclusion criteria implementation, tabulation of patient characteristics). The remaining challenges may include ensuring the use of large-scale and/or diverse data. For example, linking Diagnostic Procedure Combination (DPC) data to the NDB could enable more accurate identification of inpatient clinical events and disease severity by complementing population wide claims data with detailed hospitalization-level information, because DPC data are derived from a Japanese case-mix classification and payment system for acute inpatient care, and include standardized information on diagnoses, procedures, and resource use for hospitalized patients [14].

To advance post-marketing drug safety assessment in Japan, PMDA will prepare for expanded pharmacoepidemiological studies by leveraging enhanced data sources and advanced scientific methods and technologies, including causal inference methods and AI, and by systematically strengthening its human resource development and organizational capabilities through the accumulation of analytical experience and technical training.

More regulatory collaborations for a safety information exchange under confidential agreement with other regulatory agencies such as the US Food and Drug Administration and European Medicines Agency will also contribute to better understanding of foreign situations surrounding drug safety and early consideration of necessary safety measures in Japan.

PMDA will work continuously to optimize the post-marketing drug safety assessment process in Japan through the efforts described above. Recently, to commemorate its 20th anniversary in 2024, PMDA established a new purpose: namely, “Making everyone’s lives brighter together”. To achieve our mission, PMDA will move to the next stage with further international collaborations to provide innovative drugs promptly and safely to patients.

## Data Availability

No datasets were generated or analyzed during this work.
